# Migration of nephrostomy tube into right atrium during percutaneous nephrolithotomy: A case report

**DOI:** 10.1016/j.ijscr.2023.108759

**Published:** 2023-09-01

**Authors:** Fatemeh Esfandiari, Nasrollah Abian, Pezhman Kharazm, Amir Bigdeli

**Affiliations:** a Department of Urology, 5 Azar Hospital, School of Medicine, Golestan University of Medical Sciences and Health Services, Gorgan, Iran; b Vascular Surgery, 5 Azar Clinical Research Development Unit, 5 Azar Hospital, School of Medicine, Golestan University of Medical Sciences and Health Services, Gorgan, Iran; c Department of Nephrology and Hypertension, Sayad Shirazi Hospital, School of Medicine, Golestan University of Medical Sciences and Health Services, Gorgan, Iran

**Keywords:** Catheter migration, Percutaneous nephrostomy, PCNL, Case report, Right atrium

## Abstract

**Introduction and importance:**

Percutaneous nephrolithotomy (PCNL) is one of the most commonly performed surgeries in urology. Due to blind nature of the procedure unexpected events are inevitable. Misplacement of percutaneous nephrostomy (PCN) during PCNL into the venous system is one of the rarest complications causing great stress to both physician and the patient. Due to scarcity of data, no standard treatment has been proposed. Here, we present a case with misplaced PCN into venous system moving up to the right atrium and discuss its management with a review of the literature.

**Case presentation:**

After stone removal of a 65-year old man by PCNL, PCN was passed through access sheath supposedly into renal pelvis but it actually misplaced into venous system and traversed into right atrium. The complication was diagnosed by immediate CT scan and managed by PCN pulling back without the need to perform open surgery.

**Clinical discussion:**

Blind nature of PCNL makes it susceptible to inadvertent complications. Misplaced PCN into venous system is very rare, happening in about 13 patients worldwide. While some ended up open surgery, all of them were managed by pulling the PCN backwards. Our case is the first case in whom PCN traversed through IVC and reached right atrium during PCNL. Pulling back the PCN was a successful treatment in our case either.

**Conclusion:**

While horrifying, misplaced PCN into venous system can be managed conservatively by pulling it backwards, even if it reaches the right atrium as happened in our case.

## Highlights

• Nephrostomy tube might migrate to venous system in percutaneous nephrolithotomy.
• Threshold for CT to evaluate complications of PCNL must be low.
• Pulling back the nephrostomy tube from venous system into renal pelvis is safe.

## 1 Introduction

Since introduction of percutaneous nephrolithotomy (PCNL) by Fernström et al. [1], many advances has occurred in order to make this procedure more efficient with less complications. Yet, due to blind nature of this surgery, inadvertent complications are inevitable. Vascular complications requiring transfusion occur in up to 10 % of PCNLs [2] which rarely might happen during percutaneous nephrostomy (PCN) insertion at the end of PCNL. Here we describe a case of PCN misplacement during PCNL which traversed along renal vein into right atrium, then we discuss its management and review the literature about this complication. Our case report has been reported in line with the SCARE criteria [3].

## 2 Presentation of case

A 65-year-old man with no significant past medical or surgical history, referred to our center for left side PCNL having a 15 mm stone in the renal pelvis and a 10 mm stone in the lower calyx. After placing ureteral catheter and injecting contrast into left pyelocaliceal system (PCS), Chiba needle was inserted in the lower calyx with C-Arm guidance. Then, guidewire was passed through the needle into PCS. Tract was then dilated up to 28Fr and Amplatz was placed as access sheath. During surgery, we noticed a subtle bleeding in the operating field which was diminished by irrigation.

After removing the stones and inserting a 4.8Fr double J stent antegradely, as renal pelvis was clear with no apparent active bleeding, we pulled out the nephroscope while maintaining the access sheath. In order to place PCN, balloon port of a 16Fr foley catheter was cut and the catheter was passed through access sheath. After placing the PCN, we noticed a large outflow of blood from the PCN. So, the catheter was quickly clamped to pack the bleeding. Also, signs of tachyarrhythmia was presented on monitor but it faded away after few minutes. The access sheath was then removed and PCN was fixed to the skin by silk sutures.

**Fig. 1:**
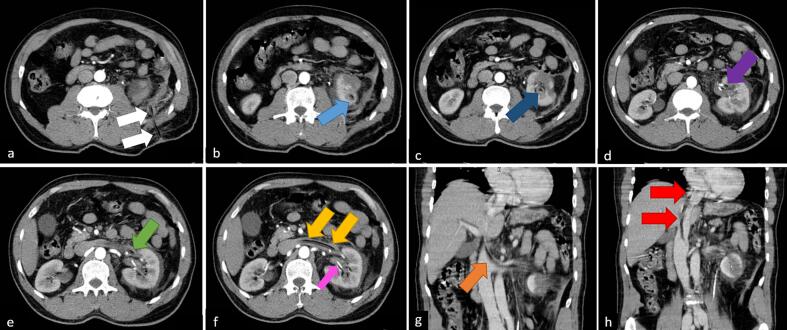
Abdominopelvic CT scan with IV contrast. a. The nephrostomy tube from skin to kidney (white arrow). b. The tube entering kidney from access tract (light blue arrow). c. The tube in renal pelvis (dark blue arrow). d, e. The tube entering renal vein (purple and green arrow). f. The tube in the left renal vein (yellow arrow), Part of double J stent in kidney (pink arrow). g. The tube traversing left renal vein and IVC (orange arrow). h. The tube entering right atrium (red arrow). (For interpretation of the references to color in this figure legend, the reader is referred to the web version of this article.)

As the patient was hemodynamically stable, he was transferred to the recovery room where we unclamped the PCN about 20 min after procedure but still pure blood came out of it. Considering possibility of vascular injury, the patient was subjected to computerized tomography (CT) of the abdomen and pelvis with and without intravenous contrast. The CT scan showed that PCN tube passed through the left renal vein into the inferior vena cava (IVC) and went up to the entrance of the right atrium, but no sign of expanding hematoma in retroperitoneum was seen [Fig. 1].

Vascular surgery consultation was done urgently. With the vascular surgeon standing by and laparotomy set in the operating room, under general anesthesia and by fluoroscopic guidance, PCN was pulled back slowly from right atrium to the IVC and left renal vein until it fell into renal pelvis. Fluoroscopic serial imaging was used to make sure about the proper PCN placement into the PCS. In order to confirm presence of PCN in PCS, contrast was injected into PCN. The contrast was seen in PCS and ureter in the fluoroscopic imaging.

Fortunately, there was no signs of active bleeding during this procedure and hemodynamic status remained stable. The patient was admitted to ICU. Intravenous antibiotic and anticoagulation therapy was administered. 1 packed RBC was injected. On post-operation day (POD) 1, CT scan was performed showing no evidence of hematoma or active bleeding. On POD 2, PCN urine became completely clear. PCN was removed on POD 3. The patient was discharged on POD 4 with a good general condition. His follow up CT scan after 3 weeks showed no signs of hematoma nor stone residue in PCS. Double J stent was removed one month after surgery.

## 3 Discussion

Since introduction of PCNL by Fernström et al. [1], many advances has occurred in order to make this procedure both more efficient and safer. Yet, due to blind nature of this procedure –at least in some parts of it- complications are inevitable. Vascular complication requiring transfusion occurs in up to 10 % of PCNLs which is generally due to initial puncture, tract dilation, and maneuvers to clear kidney stones [2]. Rarely vascular complication might happen during PCN insertion at the end of PCNL. About 13 cases of PCN misplacement into venous system have been reported worldwide; in 11 cases, PCN misplacement happened during PCNL [4,5]. All of them were in the renal vein or IVC. Only in one case, catheter was misplaced into the right atrium, which happened during PCN replacement, not PCNL [6]. All of the aforementioned cases were managed with nephrostomy removal –by pulling the tube out either percutaneously or during open surgery- without the need for vascular intervention.

Our case is the first case in whom PCN traversed through IVC and reached right atrium during PCNL. Subtle bleeding after tract dilation which resolves by irrigation is generally considered normal during PCNL. But, retrospectively looking, this finding could be due to insult to an interlobar vein. The hazardous injured site in the interlobar vein made the situation prone to misplacement of PCN tube where the tube slipped into the injured site of the vein, passed through renal vein, reaching IVC and finally right atrium. Placing PCN was used to be a standard approach during PCNL, but nowadays tubeless PCNL (insertion of DJ after PCNL) or totally tubeless PCNL (neither placing PCN nor DJ after PCNL) are much more popular. Tubeless PCNL is the standard approach in our institution. Rarely, according to surgeon's preference a nephrostomy tube might be inserted as a safety measure to improve hemostasis within the access tract and enhance urine drainage. In this patient, maneuvers to clear the stone from pyelocaliceal system concerned our surgeon thus PCN was inserted in addition to DJ.

Tachyarrhythmia after placing the PCN, faded within few minutes without specific intervention, was interpreted as a simple tachycardia due to pain or blood loss after surgery; but retrospectively looking, this could be due to stimulation of right atrium by tip of PCN catheter. On the other hand, presence of pure blood outflow through PCN immediately after its insertion during PCNL is generally normal and after few minutes, the color will turn into light red. But, presence of blood jet from PCN even after twenty minutes of clamping in the recovery room concerned us about a major vessel injury in kidney. Patient's stable hemodynamics let us perform immediate CT scan to reach the proper diagnosis i.e. PCN misplacement. Although the vascular surgeon was in the operating room, pulling the PCN back was enough to alleviate the complication with no need to perform open surgery, possibly because the injured interlobar vein was compressed by the renal parenchyma causing hemostasis.

## 4 Conclusion

Due to blind nature of PCNL, PCN misplacement into venous system -although extremely rare- might happen. Thus, each suspicious sign must be considered meticulously by the surgeon from the beginning (e.g. tract dilation) until the end (e.g. PCN outflow) of the surgery. While horrifying when diagnosed, misplaced PCN into venous system can be managed conservatively by pulling it backwards, even if it has reached the right atrium as happened in our case.

## Abbreviations

PCNL: Percutaneous nephrolithotomy
PCN: Percutaneous nephrostomy
PCS: Pyelocaliceal system
IVC: Inferior vena cava
POD: Post-operation day

## Consent

Written informed consent was obtained from the patient for publication of this case report and accompanying images. A copy of the written consent is available for review by the Editor-in-Chief of this journal on request.

## Ethical approval

This case report is exempt from ethical approval due to the nature of the

article (a description of a rare complication of a very common surgery in urology performed by endourologist and managed accordingly), as per the ethical review board at our institution.

## Funding

No funding source.

## Author contribution

Study concept: Fatemeh Esfandiari

Data collection: Fatemeh Esfandiari, Nasrollah Abian, Pezhman Kharazm

Data interpretation: Fatemeh Esfandiari, Nasrollah Abian, Amir Bigdeli

Writing the paper: Nasrollah Abian

Revision: Fatemeh Esfandiari, Pezhman Kharazm

## Guarantor

Fatemeh Esfandiari, Nasrollah Abian

## Research registration number

Although pretty rare, our case report is not “First in Man”.

## Conflict of interest statement

No conflicts of interest.
